# CapZ-lipid membrane interactions: a computer analysis

**DOI:** 10.1186/1742-4682-3-30

**Published:** 2006-08-16

**Authors:** James Smith, Gerold Diez, Anna H Klemm, Vitali Schewkunow, Wolfgang H Goldmann

**Affiliations:** 1 Friedrich-Alexander-University of Erlangen-Nuremberg Center for Medical Physics and Technology, Biophysics Group Henkestrasse 91, 91052 Erlangen, Germany

## Abstract

**Background:**

CapZ is a calcium-insensitive and lipid-dependent actin filament capping protein, the main function of which is to regulate the assembly of the actin cytoskeleton. CapZ is associated with membranes in cells and it is generally assumed that this interaction is mediated by polyphosphoinositides (PPI) particularly PIP_2_, which has been characterized *in vitro*.

**Results:**

We propose that non-PPI lipids also bind CapZ. Data from computer-aided sequence and structure analyses further suggest that CapZ could become partially buried in the lipid bilayer probably under mildly acidic conditions, in a manner that is not only dependent on the presence of PPIs. We show that lipid binding could involve a number of sites that are spread throughout the CapZ molecule *i.e*., alpha- and beta-subunits. However, a beta-subunit segment between residues 134–151 is most likely to be involved in interacting with and inserting into lipid membrane due to a slighly higher ratio of positively to negatively charged residues and also due to the presence of a small hydrophobic helix.

**Conclusion:**

CapZ may therefore play an essential role in providing a stable membrane anchor for actin filaments.

## Background

The actin cytoskeleton is a major component in determining and maintaining the shape of animal cells and is responsible for various motile phenomena. It is regulated by actin-binding proteins that are controlled by a variety of signalling molecules including the well-characterized polyphosphoinositides (PPIs). One of the capping proteins is the calcium-insensitive CapZ, which is regulated by phosphatidylinositol 4,5 bisphosphate (PIP_2_) [1-4]. This protein regulates the spatial and temporal growth of the actin filament by capping its barbed (and fast growing) end.

CapZ proteins have been isolated from various species, and sequence studies demonstrate extensive homology among *Drosophila*, *Saccharomyces*, *Dictyostelium, Acanthamoeba*, *Caenorhabditis *and vertebrates. The protein is composed of two subunits, labelled alpha and beta. The alpha-subunits range between 32 kDa and 36 kDa; the beta-subunits are generally smaller, ranging between 28 kDa and 32 kDa. To date, actin binding has only been ascribed to the beta-subunit [5], although both subunits are required for capping activity [6]. Although they show low sequence identity, alignments of the subunits reveal regions of functionally conserved residues, suggesting the presence of common motifs or putative epitopes for intermolecular binding. A structural analogy between the alpha- and beta-subunits was confirmed in a recent crystallographic study of CapZ from chicken muscle that revealed a striking resemblance in the fold of the two subunits [7].

Spatial and temporal localization studies in non-muscle cells have not always produced a consistent picture: in one case the distribution is nuclear, while chicken CapZ is concentrated in epithelial cell-cell junction complexes. Yeast capping proteins are found at the membrane in regions generally rich in actin [8]. In muscle cells, CapZ is present at the Z-line independently of actin and probably binds to other protein partners in this region [9].

Here we report that CapZ has the potential to bind to lipids (other than PIP_2_) and could therefore interact with, or embed into, lipid regions consisting of phospholipids, glycolipids, cholesterol and/or long-chain fatty acids. Our computational analysis indicates that the C-terminal half of CapZ beta-subunit could contribute to lipid interaction/insertion. CapZ may therefore play an essential role in providing a stable membrane anchor for actin filaments.

## Methods

The search for highly hydrophobic or amphipathic segments within the CapZ sequence includes the construction of plots of the average hydrophobicity and of the average hydrophobic moment [10]. The normalized 'consensus' scale of Eisenberg *et al*. [11] was taken as the hydrophobicity scale for amino acids. The number of amino acids examined together (also known as the window size) determined the type of segment under investigation.

To detect lipid membrane binding and hydrophobic motifs, and potentially antigenic regions, a window size of 11 residues was employed. The algorithm for detecting putative lipid-binding hydrophobic polypeptide sequence segments discriminates between surface-seeking and transmembrane regions. Computationally, this is performed by constructing and interpreting plots for the average hydrophobicity <H> and the average hydrophobic moment <μ_*H>*_ of selected polypeptide segments using a normalized 'consensus' scale [11-13]. According to Eisenberg *et al*. [11], various regions in a polypeptide can be divided by boundary lines, conditional on the values of <H> and <μ_*H*_>, giving three alpha-helical properties: transmembrane, lipid surface-seeking and globular. In general, transmembrane helical regions have a low <μ_*H*_> and high <H> whereas surface-seeking helical regions have a high <μ_*H*_> and average <H> [10]. In this work, we used two ratios to assay for surface-seeking propensity, *r*_*surface *_and *r*_*tm*_, relating respectively to the transition from a globular to a surface-seeking property and from a globular to a transmembrane property. These two ratios depend on <μ_*H*_> and <H>, where *r*_*surface *_= <μ_*H*_>/(0.603 - 0.392<H>) and *r*_*tm *_=*<*H>/0.51. Three conditions exist, depending on the Eisenberg plot [11]: (1) if *r*_*surface *_and *r*_*tm *_are both less than or equal to 1.0, then the polypeptide region is globular; (2) if either *r*_*surface *_or *r*_*tm *_is greater than 1.0 and the other less than or equal to 1.0, then the larger ratio determines the characteristic property; (3) if both values are greater than 1.0, then the region is said to be surface-seeking.

An amphipathic helical region was defined by the simple requirement for an effective interaction between an alpha-helix and acidic lipids. The interaction motif is suitable for amino acid segments with a length of 18 residues, which would represent five complete turns of an ideal alpha-helix. When projected on to a plane, the consecutive residues of an ideal helix are spaced with a periodicity of 3.6 at 100 degree intervals. For the amphiphatic helical analysis, a matrix incorporating information about the distribution of physico-chemically different residues was employed. This matrix also included information regarding amphiphatic structure. This approach is based on a previous treatment by Hazelrig *et al*. [14]. With an amino acid window size of 18, the results were plotted above the middle residue of the window.

Hydrophobic moments of alpha-helices and beta-strands were calculated, assuming periodicities in the hydrophobicity of 3.6 and 2.0 residues, respectively. The entire process yields several candidate sites that relate to sequence and conformational motifs for each candidate protein sequence. The two protein sequences used were obtained from the NCBI database: residues 1 to 286 from the alpha-subunit from NP006126, and residues 1 to 272 from the beta-subunit from NP004921, both from *Homo sapiens*. The lipid-binding properties of each candidate site can subsequently be evaluated using a variety of *in vitro *techniques.

Here, the experimentally-supported lipid-binding sites for *Homo sapiens *CapZ correlated with regions in the high-resolution crystal coordinates obtained from *Gallus gallus *and deposited in the Protein Data Bank (PDB code 1IZN). Over the range of sequences used there was almost 100% identity between the CapZ subunits from *Homo sapiens *and *Gallus gallus*. Molecular visualisation software packages, SPDBV and PYMOL, were used to characterize the secondary and tertiary structure, the solvent accessibility and the electrostatic field potentials [15,16]. Electrostatic calculations were performed using SPDBV using the Coulomb method, with the dielectric constant for solvent set at 80.0 and incorporating only charged residues.

## Results

The secondary structure analysis of the CapZ sequence was started with the search for segments with maximum hydrophobic and amphipathic character. The most hydrophobic segments and the most amphipathic helical segments were found in the amino-terminal region of the protein between residues 113–130 and 225–242 both in the alpha-subunit and between residues 134–151 and 215–232 both in the beta-subunit.

**Figure 1:**
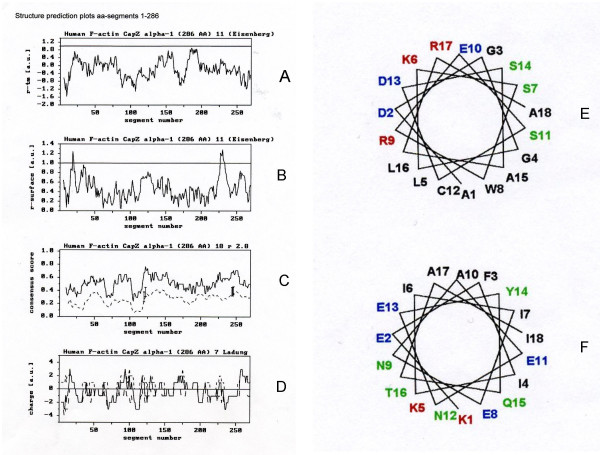
Structure prediction plots of CapZ alpha-subunit (residues 1–286) using matrix analyses according to Tempel *et al*. [10]. (A) Hydrophobicity, (B) Hydrophobic moment and (C) Probability of residues for CapZ alpha-subunit. Secondary structures (D) were calculated according to Eisenberg *et al*. [11] using a window of 11 residues. The secondary structure analyses of 113–130 (ADGGLKSWRESCDSALRA) and 225–242 (KEFIKIIENAENEYQTAI) are shown in (E) and (F), respectively. The two methods were carried out as follows: The 1^st ^method relies only on the average amino acid composition of secondary structural segments (helix, sheet, coil) in a learning set of proteins, which showed an alpha-content of 55.2%, beta-content of zero, and a coil-content of 44.8% for (E); and an alpha-content of 100% and beta- and coil-contents of zero for (F). The 2^nd ^method relies on composition fluctuations in the secondary structural segments (helix, sheet, coil) of a learning set of proteins, which showed an alpha-content of 38.1%, a beta-content of zero, and a coil-content of 61.9% for (E); and an alpha-content of 93.8%, a beta-content of 6.2%, and coil-content of zero for (F) [27-28].

**Figure 2:**
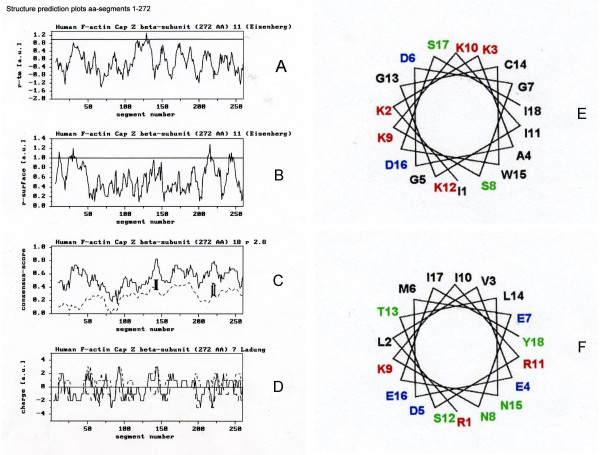
Structure prediction plots for CapZ beta-subunit (residues 1–272) using matrix analyses according to Tempel *et al*. [10]. (A) Hydrophobicity, (B) Hydrophobic moment and (C) Probability of residues for CapZ beta-subunit. Secondary structures (D) were calculated according to Eisenberg *et al*. [11] using a window of 11 residues. The secondary structure analyses of 134–151 (IKKAGDGSKKIKGCWDSI) and 215–232 (RLVEDMENKIRSTLNEIY) are shown in (E) and (F), respectively. The two methods were carried out as follows: The 1^st ^method relies only on the average amino acid composition of secondary structural segments (helix, sheet, coil) in a learning set of proteins, which showed an alpha-content of zero, beta-content of zero, and a coil-content of 100% for (E); and an alpha-content of 67.2% and beta-content of 32.8%, and coil-content of zero for (F). The 2^nd ^method relies on composition fluctuations in the secondary structural segments (helix, sheet, coil) of a learning set of proteins, which showed an alpha-content of 16.4%, a beta-content of zero, and a coil-content of 83.6% for (E); and an alpha-content of 100%, beta- and coil-contents of zero, for (F) [27-28].

Figures 1 and 2 represent the structure prediction plots calculated for the CapZ primary sequence residues 1–286 (for the alpha-subunit) and 1–272 (for the beta-subunit). The plots (a+b) of the *r*_*tm *_and *r*_*surface *_ratio profiles evaluate the hydrophobic or amphipathic alpha-helical stretches. For these calculations an amino acid window size of 11 was used. The plot in (c) represents the matrix calculations for an amphipathic alpha-helix motif. At a window size of 18 residues, the consensus score of the existing sequence (continuous line) and the average consensus score of 400 sequence randomizations (dotted line) are plotted for every segment. For any segment, the standard deviation (SD) of the randomizations is denoted by a vertical bar in the SD, where factor Γ was greater than 3.0. The quantitative distribution of charged amino acids within 7-residue segments in (d) are marked by the continuous and discontinuous lines of positively and negatively charged residues.

Results from the plots in Figures 1 and 2(a–d) from residues 1–286 for the alpha-subunit and residues 1–272 for the beta-subunit indicate two possible lipid binding regions in each: residues 113–130 and 225–242, and residues 134–151 and 215–232, respectively. Secondary structure analysis points to alpha-helical structures. No transmembrane binding domain is discernible in the alpha-subunit; therefore, the polypeptide sequence represents a helical motif with more amphipathic character. If there were lipid binding, the expectation would be near-parallel orientations of the alpha-helical axes with the plane of the membrane, so that the hydrophobic/uncharged amino acids of the alpha- subunit would interact hydrophobically with lipid chains.

Specifically, the segment 113–130 in the alpha-subunit shows a high ratio of positively and negatively charged amino acids that form the hydrophilic side of the amphipathic helix. The hydrophobic helix shows seven non-polar and three polar amino acids and would be poorly-equipped for lipid binding/insertion. The segment 225–242 in the alpha subunit, however, shows high contents of positively and negatively charged and polar amino acids, and could interact strongly with the hydrophilic (and hydrogen-bonding) side of the opposite amphipathic helix. The hydrophobic side of the helix contains six non-polar and one polar amino acid, including a strongly hydrophobic amino acid (phenylalanine, F). This gives this helix its predominantly amphipathic character. The glutamic acids (deprotonated at pH 7.0) at positions 11 and 13 would seem to make the helix unsuitable for surface binding to a negatively-charged lipid layer.

The segment 134–151 in the beta-subunit shows a slightly higher ratio of positively to negatively charged amino acids on the hydrophilic side of the short amphipathic helical region within the beta-strands, whereas the hydrophobic helical side contains seven non-polar and one polar amino acid. This distribution of positively charged amino acids would be more favourable for surface binding to negatively charged lipid layers. The segment 215–232 in the beta-subunit shows a similar amphipathic charge distribution to segment 225–242 in the alpha-subunit; however, the (negatively charged) glutamic acid at position 7 probably makes any surface binding to lipid unfavourable.

**Figure 3:**
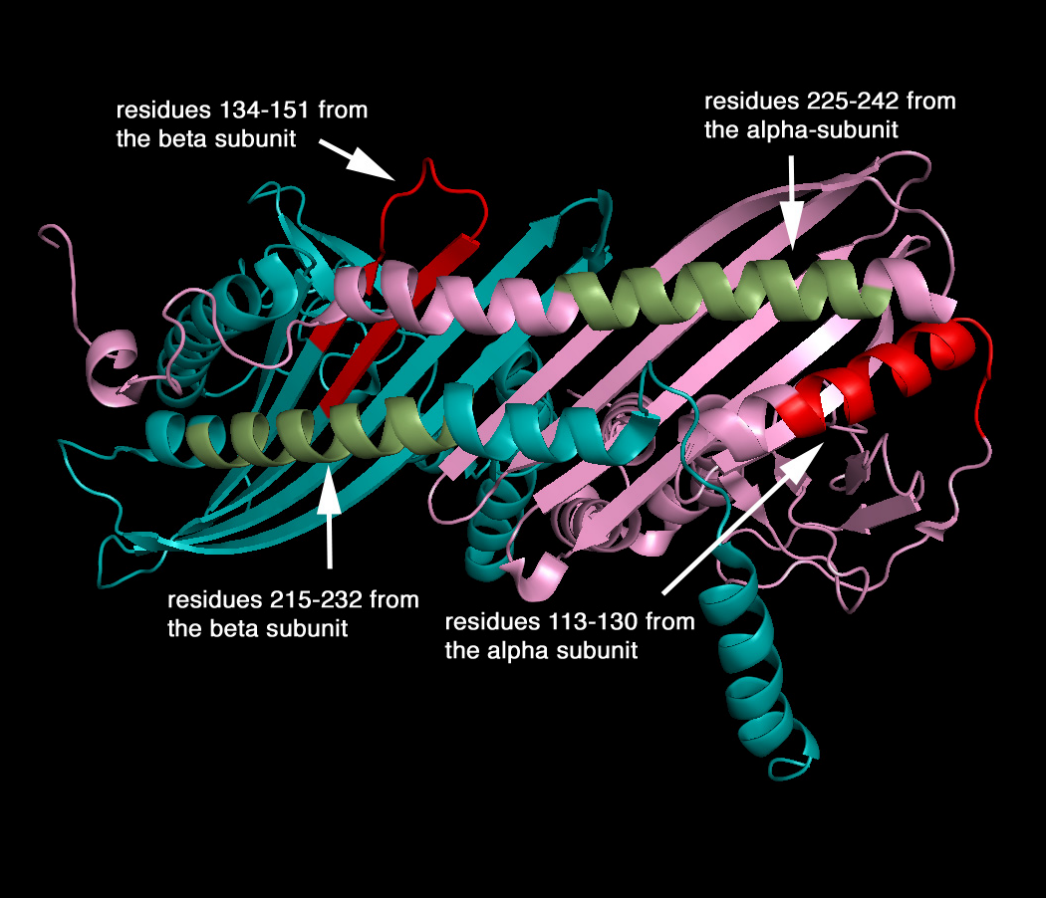
The four predicted lipid-binding sites of CapZ alpha- and beta-subunits. The coordinates of CapZ (PDB 1IZN) are displayed with the alpha-subunit shown in pink and the beta-subunit in blue. The predicted lipid-binding sites are coloured as follows: Green for the amphipathic helical regions (residues 225–242) in the alpha-subunit and (residues 215–232) in the beta-subunit; red for the amphipathic helical region in alpha-subunit (residues 113–130) and also for the putative lipid membrane inserting region within the beta-subunit (residues 134–151).

The recent crystal of CapZ shows two subunits that are structurally analogous creating a pseudo-two-fold symmetry perpendicular to the long axis of the molecule (Figure 3). Each subunit contains three domains and an additional carboxyl-terminal extension. Three anti-parallel helices (helices 1–3) that form the amino-terminal domain are in an up-down-up arrangement. The middle domain is composed of four beta-strands (strands 1–4) for the alpha-subunit and three (strands 1–3) for the beta-subunit, containing two reverse turns. The carboxyl-terminal domain comprises an anti-parallel beta-sheet formed by five consecutive beta-strands (strands 5–9), flanked on one side by a shorter amino-terminal helix (helix 4) and a long carboxyl-terminal helix (helix 5). The beta-strands of each subunit form a single 10-stranded anti-parallel beta-sheet in the centre of the molecule. The sequence implicated in lipid binding, amino acid residues 134–151 in the beta-subunit, forms largely beta-sheet that is probably flexible and solvent-accessible despite contributing residues to the strong dimer interface (for example, *via *lysine 136).

## Discussion

Recently, it has been reported that when gelsolins (calcium-dependent actin-binding proteins) are presented with high lipid concentrations they can bind as many as ten PtdIns(4,5)P_2 _molecules [17]. The value of the molar ratio between gelsolin and PtdIns(4,5)P_2 _has been contentious, complicated by differences between studies in the state or presentation of the lipid. However, when presented as a minor component with other lipids (*i.e*. cholesterol), one PtdIns(4,5)P_2 _binds one gelsolin, close to the physiological situation of 0.3–1.5%, which then allows it to associate with the plasma membrane [18].

Furthermore, it has been reported that polyphosphoinositides (PPI) form aggregates within the bilayer under the influence of certain proteins [19] and there may be many possible modes of binding to PPI and other lipids. The finding that several sites within gelsolin can be cross-linked to PPI analogues would seem to support this view [20]. Together with our present data, indicating that CapZ could bind non-PPI lipids with high affinity, it seems likely that CapZ may bind up to four PtdIns(4,5)P_2_, if they are available, through direct hydrogen-bonding interactions with the binding sites; however at lower PtdIns(4,5)P_2 _concentrations these sites may be occupied by other lipids. This is in agreement with observations by differential scanning calorimetry, film balance and spectroscopy, which have shown that proteins require a net negative charge created by lipids other than PPIs, a hydrophobic interface or indeed PPI for membrane interaction/insertion [17].

CapZ has been found to be associated with both membranes and actin filaments in activated macrophages and platelets [21,22]. This is a surprise since PtdIns(4,5)P_2 _has been assumed to be the binding partner of CapZ and yet this lipid dissociates the CapZ-actin complex [23,24]. It is possible that the binding sites for the CapZ-actin complex in macrophages and platelet membranes are lipids other than PPIs and that these do not dissociate the complex. It has been reported that binding of gelsolin or indeed filamin (a dimeric actin cross-linking protein) to phosphatidylglycerol/phosphatidylcholine small unilaminar vesicles does not inhibit the nucleation of actin polymerization or cross-linking.

This work raises the possibility that CapZ not only binds to the lipid surface, but also becomes partially embedded within the lipid bilayer due to the residues 134–151 of its beta-subunit. Previous studies have indicated that various peptides derived from PPI-binding regions of, for example gelsolin, Arp2/3, talin *etc*. have this capacity in isolation [25]. The authors have also found that such peptides can incorporate into phosphatidylglycerol/phosphatidylcholine small unilaminar vesicles in the absence of PPIs [25]. The importance of hydrophobic interactions between these proteins and PPIs has been suggested by molecular dynamics studies in which the PPIs are to some extent pulled out from the bilayer [26].

In conclusion, a number of sites in CapZ have been proposed to bind lipids and these tend to be located in linker regions between the discrete domains of the protein. The main sites appear to be in the linker regions, 134–151 and 215–232 in the beta-subunit and secondary sites have been identified within the alpha-subunit. We suggest further that the first region 134–151 in the beta-subunit becomes inserted between lipid heads and perhaps into the core of a lipid bilayer.
